# Posterior Midline Interspinal Fixation (PMIF) to Treat Persistent Severe Pain Due to Severe Compression Fracture of Thoracic Vertebral Body: A Case Report

**DOI:** 10.7759/cureus.36159

**Published:** 2023-03-14

**Authors:** Soubrata V Raikar, Arun A Patil

**Affiliations:** 1 Pain Management, Midwest Anesthesia Pain Management, Elkhorn, USA; 2 Surgery, Creighton University School of Medicine, Omaha, USA

**Keywords:** interspinal distraction fixation, percutaneous vertebroplasty, kyphoplasty, intractable pain, thoracic compression fracture

## Abstract

Generally, severe persistent pain due to compression fractures of the lumbar and thoracic vertebral bodies in the elderly, that fail conservative measures are treated with vertebroplasty or kyphoplasty. However, in the case reported in this paper, the compression fracture was so severe, that accurate bone needle placement into the vertebral body was felt to be difficult. In addition, there was a high risk of extravasation of the cement into the surrounding structures or blow-up of the lateral wall of the vertebral body. Therefore, a simple operation of posterior midline interspinal fixation (PMIF) was performed.

The patient was a 91-year-old lady with severe pain in the mid-thoracic spine due to a severe compression fracture of the seventh thoracic vertebral body that was totally flattened in its anterior part. The patient was neurologically intact. However, she had difficulty walking, because the pain was very severe in an upright position. She was treated with a back brace and oxycodone for six weeks without any benefit.

Because she was a poor candidate for vertebroplasty or kyphoplasty, a PMIF system was implanted. Postoperatively, within two weeks, her pain score dropped from 9/10 to 0/10; and from two months onwards she was completely free of pain medications until her death from an unrelated cause, 18 months after the operation.

This is the first reported case of PMIF for the treatment of pain due to vertebral body compression fracture in the elderly. PMIF is a simple minimally invasive procedure without compromising the facet or any bony structure. The risk of severe complications, therefore, is remote. The success in this single case, therefore, begs for further exploration of this method in the treatment of compression fractures in the elderly.

## Introduction

Vertebral body compression fractures in the elderly are fairly common. Many of them go unnoticed because they are mild and asymptomatic. Some do have symptoms. They can be treated conservatively with a brace and pain medications. Those with severe pain who fail conservative measures may need invasive intervention. The most common procedures include vertebroplasty and kyphoplasty [1-5]. Both these procedures are done under local anesthesia with sedation using a bone needle. The needle is percutaneously inserted into the vertebral body through the pedicle under fluoroscopic guidance. Though these procedures are easy to perform, they can be challenging if the vertebral body is flattened, because it may be difficult to insert the needle toward the target. In addition, there is an increased risk of extravasation of the cement into the surrounding tissue, the creation of lateral wall burst in the fracture, and lung embolization through the venous channels [6,7].

Posterior midline interspinal fixation (PMIF) is a procedure that is gaining acceptance in the last 15 years [8-12]. It is primarily used for treating radicular pain and neurogenic claudication due to mild to moderate disc bulge, mild to moderate spinal stenosis, and foraminal narrowing. It is a minimally invasive outpatient procedure, which does not violate the spinal canal or the facets; and requires minimal tissue dissection. Though the initial devices invented were aimed at distracting the interspinal space, the more recent ones also offer rigid fixation of two spinal segments.

In this paper, a case report is presented in which a PMIF system was used to treat intractable pain, secondary to a vertebral body compression fracture in the mid-thoracic spine. This procedure was used because the patient was not suitable for other available procedures. Based on a literature search on PubMed, this is probably the first reported case of using this procedure for the treatment of pain due to compression fracture.

## Case presentation

A 91-year-old lady presented with severe sudden onset pain in the mid thoracic spine while she was walking. The pain was worse on standing, walking and bending over. The pain was relieved when she was recumbent. She did not have any neurological symptoms. Because of the severity of her pain, she was started on oxycodone and a back brace was applied. Prior to her operation she was receiving 10 mg of oxycodone 6 times a day. Even with the medication and the brace she still had significant pain. Her past history was negative for any serious illness except for prior compression fractures in the lumbar vertebral bodies, that were treated with vertebroplasty procedures. She was on medications for heart burns and mild dementia.

Physical examination

The patient was in significant distress due to the pain. She was alert and oriented. Cranial nerves were intact. She was slightly bent over when she walked. Her coordination and deep tendon reflexes were normal. She had good strength in the lower extremities. Planters were down going. She had severe tenderness in the mid-thoracic spine with minor gibbous formation. Her pain score was 9/10.

Imaging studies

**Figure 1:**
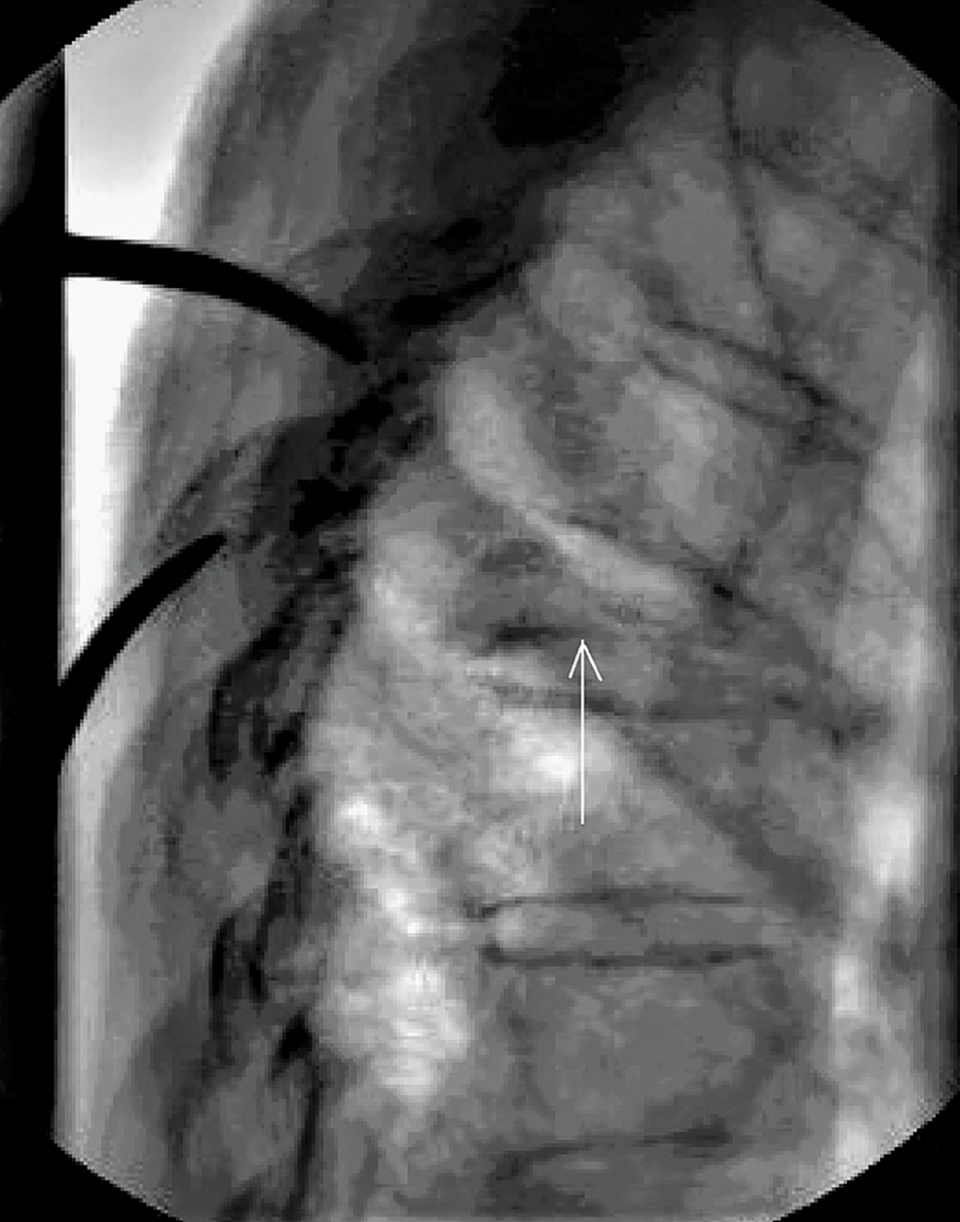
Lateral thoracic spine x-ray picture shows compression fracture (arrow) of seventh thoracic vertebral body.

The studies showed a severe compression fracture of the seventh thoracic vertebral body, with almost completely flattened in its anterior part (Figure 1).

Description of the device

**Figure 2:**
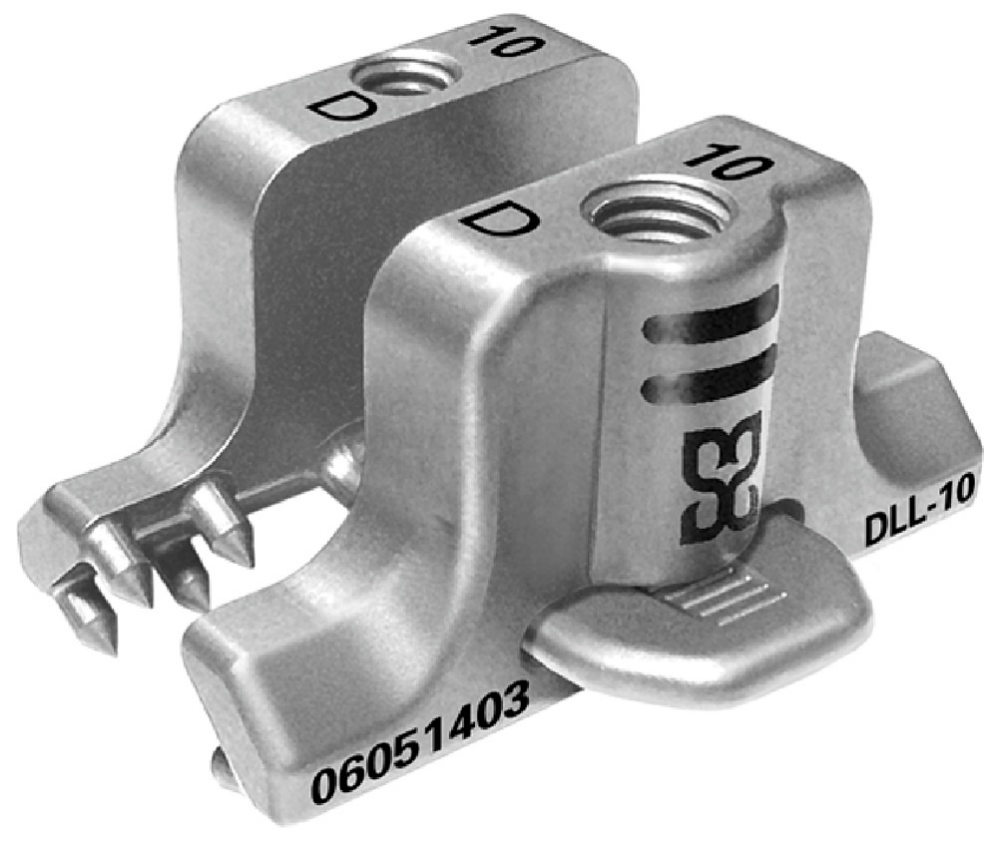
A photograph of the implant used for posterior interspinal fixation.

Stabilink Interlaminar fixation system (SIFS) (Southern Spine LCC, Macon, GA) was used (Figure 2) for the PIMF. The system has a “Laminar Lock” design and forms a 90° flare to have a stronger hold on the laminar bone. There are 16 spikes per implant over a wide area for increased load sharing. Furthermore, it has torque controlled locking mechanism.

Surgical procedure

**Figure 3:**
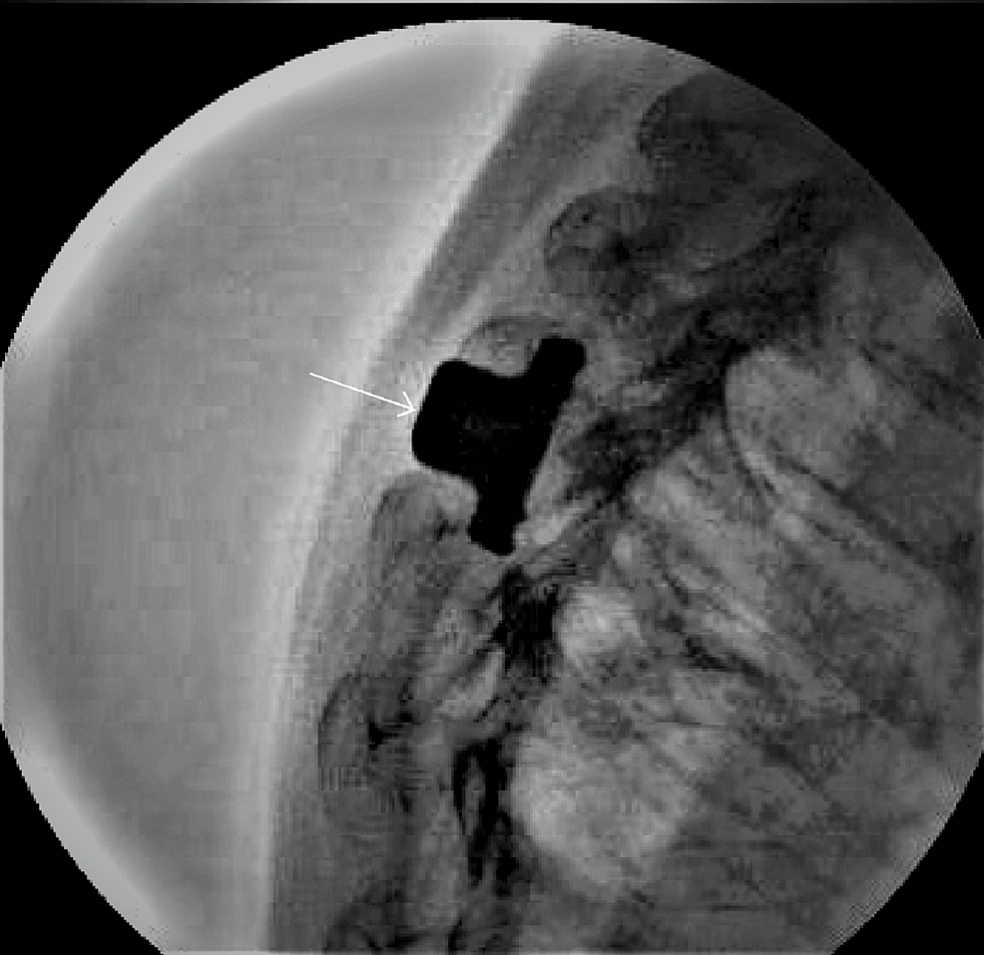
The implant (white arrow) between the thoracic sixth and seventh spinous processes.

The procedure was done in an outpatient surgery center; and patient was discharged home on the same day after surgery. The procedure was done in prone position using local anesthetics and heavy sedation. In addition, the procedure was done using fluoroscopic imaging. A midline 3-centimeter incision was made. The paraspinal muscles were separated from the posterior neural arch of thoracic sixth and seventh vertebrae. The interspinous ligament was excised deep down to the ligamentum flavum. Bony margins of the spinous processes in the interspace were roughened. The interspinal space just above the level of the laminae was measured. An appropriate size device was then inserted between the spinous processes deep down until the spikes of the device reached the laminae. The wings of the device were then compressed into the bone. The system was then locked in position (Figure 3). Demineralized bone matrix (NuFuze, Nu Tech Spine and Biologics, Birmingham, AL) was then placed between the roughened surfaces of the spinous processes. The wound was then closed.

Results

There was no complication from the operation. The blood loss was minimal. The patient reported immediate relief of severe pain after her operation. At two weeks after the operation, her pain score dropped to 0/10, was pain free even in upright position, and she stopped wearing the brace. At two months after operation, she was completely free of all pain medications until her death 18 months after the operation, from an unrelated cause.

## Discussion

Compression fracture pain is usually local. It is produced by severe strain on ligaments holding the middle and posterior column of the involved vertebra and the segment above it, and on the facet joint between them. The strain occurs when the patient is upright and while walking, from the weight of the upper part of the body on the injured segment. In the recumbent position, the patient had relief from the pain because the offended structures mentioned above were relaxed. Therefore, by fixing the vertebral segments in a recumbent position, the relaxed state of the offended structure was permanently maintained. This gave the patient relief from the pain after surgery even when she was in an upright position and walking. The procedure is simple and minimally invasive. It was done in an outpatient surgery center, and the blood loss was minimal. None of the normal bony structures or joints were disrupted; thus, reducing postoperative pain or complication. The segment above the fracture was included in the fixation because it is the major player in putting stress on the inter-segmental structures.

In recent years there is increased use of PMIF to treat lumbar spinal stenosis, foraminal stenosis, and mild to moderate lumbar disc protrusion, with good outcomes [8-12]. The success is attributed to inter-spinal distraction resulting in stretching the in-buckled ligamentum flavum, the opening of the neural foramina, and the reduction of intradiscal and interafacetal pressure. The system also rigidly fixes two spinal segments because it has strong spikes that hold the laminae and spinous processes firmly in the distracted position. In this case, being reported in this paper the device was used only for rigid fixation.

 In elderly patients with osteoporosis, posterior lateral instrumented fusion is usually avoided in the treatment of severe pain due to vertebral body compression fracture, because a major procedure can put a severe strain on the patient's vital functions. In addition, their osteoporotic bones may not be strong enough to firmly grip the pedicle screws. Vertebroplasty or kyphoplasty, therefore are generally the main procedures of choice; though in the present case, because of the reasons already discussed, these options were ruled out. Though both these procedures are fairly safe and minimally invasive, they have some disadvantages. They include the risk of extravasation of the injected cement into the surrounding structures, including the epidural space. In addition, there is a risk of the cement embolus going to the lung via the venous system [6,7]. Furthermore, there are reports of fractures in the adjacent segment after vertebroplasty and kyphoplasty [13]. On the other hand, PMIF does not have the risk of any major complication, because the procedure does not violate the spinal canal or the facets, and minimal tissue dissection is required.

## Conclusions

In conclusion, the authors present a case of severe pain due to a severe thoracic vertebral body compression fracture, which was successfully treated with a simple and minimally invasive procedure of PMIF. Since this is a single case report with no prior report of such treatment for this condition, no conclusion can be made regarding the efficacy of this procedure. However, because of the simplicity and safety of this procedure, it may be worthwhile exploring its use, as a treatment modality for pain due to vertebral body compression fractures in the elderly.
